# Battling adhesions: from understanding to prevention

**DOI:** 10.1186/s42490-019-0005-0

**Published:** 2019-02-27

**Authors:** Héctor Capella-Monsonís, Stephen Kearns, Jack Kelly, Dimitrios I. Zeugolis

**Affiliations:** 1 Regenerative, Modular and Developmental Engineering Laboratory (REMODEL), Biomedical Sciences Building, National University of Ireland Galway (NUI Galway), Galway, Ireland; 2 Science Foundation Ireland (SFI) Centre for Research in Medical Devices (CÚRAM), Biomedical Sciences Building, National University of Ireland Galway (NUI Galway), Galway, Ireland; 3 University Hospital Galway, Galway, Ireland

**Keywords:** Postoperative adhesions, Surgery, Antiadhesion barrier, Antiadhesion agents

## Abstract

Adhesions represent a major burden in clinical practice, particularly following abdominal, intrauterine, pericardial and tendon surgical procedures. Adhesions are initiated by a disruption in the epithelial or mesothelial layer of tissue, which leads to fibrin adhesion sites due to the downregulation of fibrinolytic activity and an increase in fibrin deposition. Hence, the metabolic events involved in tissue healing, coagulation, inflammation, fibrinolysis and angiogenesis play a pivotal role in adhesion formation. Understanding these events, their interactions and their influence on the development of post-surgical adhesion is crucial for the development of effective therapies to prevent them. Mechanical barriers, antiadhesive agents and combination thereof are customarily used in the battle against adhesions. Although these systems seem to be effective at reducing adhesions in clinical procedures, their prevention remains still elusive, imposing the need for new antiadhesive strategies.

## Background

Adhesions represent a major postoperative complication, particularly in abdominal, pelvic, pericardial and tendon surgical procedures, where they cause pain, stiffness and loss of function. Adhesions occur through inflammation and coagulation processes, triggered by surgery, injuries or irritation, that damage the cell monolayer placed on the basement membrane in tissues, leaving them exposed to fibrin deposition that leads to further fibroblast attachment and vasculature generation. These issues, together with a decrease in fibrinolytic activity, result in the deposition of organised extracellular matrix (ECM) and adhesion formation. The incidence of adhesions after abdominal surgery varies from 55 to 66%, and adhesions are typically underestimated by surgeons. In 1994, the estimated total financial cost of adhesions in the US was US$ 1.3 billion [1]. Intrauterine adhesions or Asherman syndrome may also reach a prevalence of 45% [2]. Pericardium adhesions contribute to an increase in the rate of inadvertent injuries, which is approximately 7 to 9% [3], and have been estimated to increase due to the growing number of cases of cardiac reoperations [3, 4]. Moreover, adhesion formation is a major problem in tendon repair, which entails a loss in the range of motion in the flexor tendon from 16 to 27% of cases [5] and reoperation in 4% of cases [6].

Adhesions can be classified into de novo, which originate in a tissue area for the first time, and secondary adhesions, which are produced in areas that adhesions had previously formed. As a function of their location, structure and derived pathology, adhesions can remain silent or cause complications [7, 8]. The severity of the complications caused may vary depending on the tissue where they are located. For instance, in abdominal surgery, adhesions may lead to abdominal pain and small bowel obstruction, whereas in pelvic surgery, they may lead to female infertility [1]. Additionally, pericardial adhesions may contribute to an increase in the risk of inadvertent injuries in the heart and great vessels and perioperative bleeding [3]. Pericardial adhesions may extend the operation time, increasing the associated risk and costs. The formation of adhesions in tendon repair may involve a loss of healthy biomechanical and gliding properties, thereby limiting the function of the repaired tendon [9]. Overall, adhesions result in pain, loss of tissue function and severe complications. Thus, the development of preventive systems that avoid the formation of adhesions is crucial to improve surgical outcomes and reduce patients’ pain, reoperation rates and subsequent costs. Although different methods are currently employed, adhesion prevention formation remains a major challenge in surgery. Thus, further efforts are needed to develop an efficient system that will prevent the formation of post-operative adhesions.

In this review, we aim to describe the mechanism underlying adhesion formation, including the pathways, metabolites and cell types involved. The specific characteristics of adhesions in the different tissues will also be identified. Different methodologies that are currently being investigated and used to battle adhesion formation will also be discussed.

## Adhesion formation and metabolic pathways

**Fig. 1:**
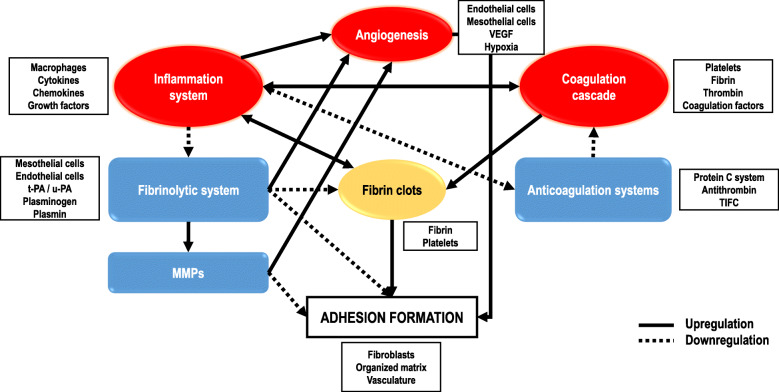
Interaction of the main pathways involved in adhesion formation. Inflammation, which is mainly mediated by macrophages, through cytokines and growth factors initiates the healing process by promoting fibrin exudate formation. Coagulation is simultaneously elicited, resulting in the formation of fibrin clots, which amplify the inflammatory response and attract inflammatory cells. When coagulation occurs, the anticoagulation system activates to downregulate the formation of thrombin and subsequently, fibrin. Both inflammation and anticoagulation downregulate each other. The fibrinolytic system degrades fibrin and extracellular matrix (ECM) components; however, inflammatory cytokines regulate the formation of plasminogen activator inhibitors (PAI), downregulating fibrinolytic activity. Fibrinolytic system components can activate MMPs, which are in charge of degrading ECM components. Both MMPs and the fibrinolytic system are involved in angiogenesis, the production of new vasculature that is promoted by inflammatory cytokines and hypoxia. Hypoxia occurs in fibrin clots, sparking the differentiation of fibroblasts to adhesion phenotype fibroblasts. This process, together with angiogenesis, promotes the deposition of organised ECM and subsequently, adhesions

Adhesion formation results from an imbalance between fibrin deposition and fibrinolytic activity. These events are regulated by different systems and pathways (e.g. inflammation, coagulation and fibrinolysis) that involve different cell types, their interactions and complex molecular mechanisms, which are shown schematically in Fig. 1. Understanding how these pathways work and interact is necessary to understand adhesion formation.

### Inflammatory response and coagulation mechanism

Surgery, infection or irritation lead to the disruption of the epithelial or mesothelial layer that rests on the basal membrane, leaving it exposed. Subsequent infiltration of blood neutrophils and monocytes sparks inflammation, which elicits the secretion of fibrin-rich exudate as part of the initial healing process [10]. Simultaneously, coagulation and platelet aggregation are initiated to avoid excessive blood loss, which occurs through the activation of pro-coagulation factors in the blood or the cell membrane of injured cells, platelets and vascular endothelial cells. These factors culminate in the formation of fibrin monomers from fibrinogen, which is mediated by thrombin [11, 12]. Then, the aggregation of these fibrin monomers and activated platelets form coagulation clots [12]. The pool of factors (e.g. growth factors, cytokines, chemokines, eicosanoids, and proteases) released from activated platelets, together with the degradation products of fibrinogen and fibrin cleaved by thrombin and plasmin, serves as chemo-attractants for macrophages, neutrophils, T cells, mast cells, mesothelial cells and epithelial cells that are involved in the inflammation process and in healing [13].

Chemokines are a group of polypeptides that mediate the chemo-attraction of leukocytes. The main chemokines involved during the inflammatory response are produced by macrophages [13], platelets, mesothelial cells and endothelial cells [14, 15]. Other key cytokines involved in the regulation of inflammation are TNF-α, IL-1, IL-6 and IL-12 (pro-inflammatory), as well as IL-10 and TGF-β (anti-inflammatory) [16]. These cytokines also regulate the coagulation cascade, where IL-6 mainly induces the expression of tissue factor, initiating the coagulation cascade and culminating in fibrin deposition. Reciprocally, the coagulation proteases can modulate inflammation by stimulating the production of cytokines and growth factors when they bind to protease-activated receptors (PARs), which are present on endothelial cells, mononuclear cells, fibroblasts and platelets [11].

The protein C anticoagulation pathway is another process that can influence inflammation and coagulation systems. The protein C pathway regulates thrombin formation and is triggered when thrombin binds to thrombomodulin on the membranes of endothelial cells. Then, this binding increases protein C activation and blocks the thrombin catalysis of fibrin formation and downregulates coagulation [17]. However, during inflammation, the protein C anticoagulation pathway is downregulated [11, 17]. Alternatively, anticoagulant pathways such as the protein C pathway also downregulate inflammation by blocking cytokine production and tissue factor expression [11].

### Fibrinolytic system

The fibrinolytic system is composed of the following key elements: plasminogen / plasmin, plasminogen activators (PA) t-PA and u-PA, plasminogen activator inhibitors (PAI) and plasmin inhibitors (mainly α2-antiplasmin). This system is also tightly associated with matrix metalloproteinase (MMP) activity [18].

Plasminogen is an inactive proenzyme that is converted into the active form (plasmin) by tissue plasminogen activator (t-PA) and urokinase plasminogen activator (u-PA); these reactions are inhibited by PAIs. Plasmin cleaves ECM components and efficiently degrades fibrin and is simultaneously inhibited by α2-antiplasmin [10, 18]. The components of the fibrinolytic system are produced in macrophages, wound cells and mainly mesothelial cells, which suggests a crucial role of an intact mesothelium for fibrin degradation and limitation of adhesion formation [13].

t-PA has low activity in the absence of fibrin, but in its presence, t-PA is highly effective at activating plasminogen [10, 18], and it mainly proceeds from mesothelial and endothelial cells [19]. u-PA enhances plasmin production when it binds to its receptor (u-PAR) by activating plasminogen and u-PA seems to regulate extracellular proteolytic activity [18]. However, the activation of plasminogen is further inhibited by the delayed release of PAI-1 from endothelial and mesothelial cells [11, 18]. This process is regulated by the proinflammatory cytokines TNF-α and IL-1β, and the expression of these cytokines is also stimulated by fibrin and fibrinogen [11].

Several components of the fibrinolytic system may also interact with MMPs, which main role is the degradation of ECM components. Plasmin activates some MMPs in vitro, while in vivo, the activation of certain factors is dependent on plasmin, such as plasmin-dependent activation of proMMP-9 [18, 20]. MMP-3 may also regulate the plasminogen/plasmin pathway by decreasing the amount of plasminogen available for its activation and by simultaneously inhibiting α2-antiplasmin and PAI-1 [21]. In addition, adhesion fibroblasts present elevated expression of MMP-1 and tissue inhibitor of metalloproteinase 1 (TIMP-1) [22].

In summary, coagulation and inflammation are parallel processes that maintain a tight interaction and generally promote adhesion formation since they promote the deposition of fibrin. However, the deposition of organised matrix to form adhesions also depends on the anticoagulation and fibrinolytic systems, which decreases the deposition of fibrin and metabolises the deposited matrix, respectively. Both pathways also maintain close interaction with inflammation and coagulation processes in which several mediators from each system can modulate the performance of another. These interconnections between pathways complicate the understanding of healing and adhesions but also offer a multitude of clinical targets to battle adhesion formation after surgery.

### Angiogenesis

Angiogenesis is highly involved with inflammation and fibrinolytic processes; therefore, it must influence adhesion formation. Angiogenesis comprises the degradation of the surrounding ECM and the migration and proliferation of endothelial cells to form new vascular conduits. Several cytokines and growth factors, including IL-1, IL-8, TNF-α, VEGF and TGF-β, are considered stimulators of angiogenesis [13, 23, 24]. However, angiogenesis is dependent on the balance between its promoters and inhibitors, which include numerous cytokines, growth factors and other agents [13]. In addition to endothelial and mesothelial cells, diverse cell types, such as platelets [25], macrophages [26] and fibroblasts [27], play a key role in angiogenesis. Angiogenesis starts during inflammation and requires the fibrinolytic system to initiate the invasion of endothelial cells. Furthermore, under low oxygen tension environments, adhesion fibroblasts increase the production of VEGF, promoting the formation of capillaries [24]. The growth factors that promote angiogenesis modulate the expression of fibrinolytic components and inhibitors, where the TGF-β pathway acts as a mediator [13, 23]. Although the precise mechanism by which angiogenesis modulates adhesions is not fully understood, it is believed that it has a direct effect on enhancing adhesion formation. It has been suggested that excessive angiogenesis increases the recruitment of pericytes, which adopt a fibroblastic phenotype supporting scar formation. Also, the regression of capillaries results in endothelial apoptotic cells, which presence also enhances fibrosis [28]. Nonetheless, the importance of angiogenesis for the formation of adhesions represents additional targets to develop strategies in the prevention of their formation after surgery.

## Adhesion pathophysiology

Adhesion formation is initiated by an imbalance of fibrin deposition, which is triggered by coagulation, inflammation and fibrin degradation. These processes are mainly regulated by the fibrinolytic system of endothelial or mesothelial cells. Once fibrin clots are formed, if they persist, they serve as a scaffold for the inflammatory cells and fibroblast attachment that, together with vasculature formation, lead to the deposition of organised matrix and subsequent adhesions. The permanency of the fibrin clots mainly depends on the integrity of the mesothelium and basal membrane; when these structures are compromised, fibrinolytic activity is not balanced with fibrin deposition [8, 10, 13, 15, 22]. Although this model is generally accepted for most tissues, cell populations, molecular pathways and tissue-specific complications determine in the end how adhesions will be formed.

### Peritoneum

In normal wound healing, fibroblasts undergo apoptosis, creating low oxygen tension. Subsequently, more fibroblasts attach and change their phenotype to myofibroblasts and remodel the tissue [29, 30]. Regarding peritoneal adhesions, fibroblasts change their phenotype to an adhesion phenotype under hypoxic conditions [22], upregulating the production of VEGF to enhance the reoxygenation of hypoxic tissue that these clots represent [24]. The adhesion phenotype is characterised by an increase in the expression of fibronectin, collagen type I and III in comparison to that of normal fibroblasts [22], mainly promoted by TGF-β [23]. The proteolysis of the deposited ECM carried out by fibrinolytic and MMP systems is also crucial to determine the fate of adhesions at this stage [13]. The main function of the peritoneum is to provide a frictionless and protective barrier to isolate and allow movement of organs and tissues and adhesions may interfere with these functions, causing bowel obstruction and chronic abdominal pain [31].

### Pericardium

Adhesions formed in the pericardium have also been widely studied. However, these studies are limited to the intraoperative period due to the nature and severity of the surgical procedure. The mechanism in the perioperative period has been inferred from experimental animal studies and peritoneal adhesions [3]. As observed in the peritoneum, the detachment pericardial mesothelial cells (PMC) is crucial in the further formation of adhesions since a decrease in fibrinolytic activity can be observed in the areas where denudation has occurred [3, 32]. The detachment of PMCs reportedly occurs after 135 min of pericardiotomy when they remain floating in the pericardium cavity [3, 33]. If the basal membrane remains exposed, fibrin deposition occurs, and the adhesion formation process starts and develops in the pericardium. Mediators of the inflammatory response, such as TGF-β, promote the detachment of PMCs and the loss of the epithelial phenotype for a fibroblastic one, promoting fibrotic processes [34] .The detachment of PMCs has also been related to a decrease in the activation by plasminogen [32]. During the regeneration of the mesothelium, these denuded areas may be covered by mesothelial cells from different sources, including activated mesothelial cells adjacent to the site of the injury and pre-existing floating PMCs [3, 15, 32]. When adhesions are formed on the pericardium, they may complicate contraction movements and flow, leading to several complications, including (in ascending order of severity) an increased risk of inadvertent injuries and reoperation [3], increased intraoperative bleeding [35], compression of the heart [36] and malfunction of ventricle contraction [37].

### Tendon

Tendon healing is similar to healing processes observed in other tissues [38]. Tendon healing is normally divided into the following phases: the inflammation stage where inflammatory cell recruitment occurs; the proliferation stage where tenocytes and macrophages direct the deposition of the initial matrix, mainly collagen type III; and the remodelling stage when reorganisation of ECM is carried out, and aligned collagen type I fibres are deposited [9, 39–41]. During the inflammatory phase, the infiltration of surrounding fibroblasts, commonly known as extrinsic healing, leads to the formation of adhesions. However, the repair modulated by endotenon and epitenon tenocytes results in proper healing, prevention of adhesion formation and preservation of the gliding properties of the tendon [9, 40]. Similar to the peritoneum and pericardium, the attraction and attachment of surrounding fibroblasts is initially triggered by fibrin clots in the tendon [39]. Recent research in mice has demonstrated that tendons are covered by a basement membrane and epithelium that retain cells in the tendon. When both the epithelium and basal membrane remain intact, fibrin deposition and subsequent adhesions are prevented [42]. These findings match the model of adhesion formation in other tissues that have been more thoroughly investigated. The role of macrophages can also determine the outcome of healing, where the imbalance between M1 (proinflammatory) and M2 (anti-inflammatory) macrophages can lead to poor healing or excessive tissue deposition [40]. Adhesions in tendons obstruct extension and contraction movements since they increase the friction, resulting in a loss of gliding properties and range of motion, which increases the recovery time and may cause substantial morbidity [39].

### Uterus

After pelvic surgery, the formation of intrauterine adhesions is directly related to trauma and denudation of the endometrium, which promotes the attachment of surrounding tissue [43]. More specifically, the disruption of the endometrium and exposure of the basement membrane, myometrium or connective tissue leads to the formation of scar tissue [44]. The formation of adhesions in the uterus is triggered in chronic inflammatory conditions, such as endometriosis due to the maintenance of inflammation by macrophages after the acute inflammatory response contributes to the formation of adhesions, as supported by in vivo models [45]. The insufficient re-vascularisation of the endometrium, which in normal conditions occurs cyclically with menstruation, prevents the repair of the endometrial cell layer and enhances adhesion formation, suggesting that angiogenesis in endometrium may influence its repair once adhesions are formed [46]. Moreover, in the case of intrauterine adhesions, oestrogen seems to play a crucial role, interacting with important molecules in inflammation and angiogenesis such as TGF-β or VEGF [46]. Circulating levels of oestrogen are closely related to endometrium regeneration where a decrease in its levels slows its formation by endometrial progenitor cells. This process, together with inflammation and/or infection, enhances the formation of fibrotic tissue [47]. The formation of intrauterine adhesions has a high incidence and can cause chronic abdominal pain infertility.

## Battling adhesions

The formation of adhesions is a common complication in different surgeries and interferes with the function of the tissue where they are produced. Furthermore, adhesions lead to complications of varying severities, challenging the welfare of the patients. Thus, it is crucial to limit their formation.

### Surgical procedures

The main factor that promotes the formation of adhesions after surgery is the disruption of the epithelium or mesothelium and basal membrane structure, which has been related to lower fibrinolytic activity. Thus, it is logical to assume that compared with more invasive procedures, less invasive surgical techniques that consequently reduce the damage to the epithelium / mesothelium will reduce adhesion formation.

In abdominal surgery, several studies have shown a reduction in adhesion formation with a reduction in invasiveness. For example, compared with open surgery, laparoscopy has been associated with a reduction in the formation of adhesions and their severity [1, 48]. However, other studies conflict with this idea, stating that laparoscopy has no beneficial effect on adhesion formation [49, 50]. These conflicting results could be explained by the fact that although laparoscopy reduces trauma, the desiccation, use of foreign bodies and insufflation of CO_2_ during laparoscopy can promote adhesion formation due to the induction of hypoxia [49, 51]. Similarly, the use of minimally invasive techniques that reduce PMC loss and the damage to the mesothelium is advocated to avoid pericardium adhesions; however, no evidence supporting this theory has been reported to date [3]. Alternatively, minimally invasive surgical techniques have been shown to reduce adhesion formation in Achilles tendon repair [52]. Furthermore, specific suturing techniques seem to influence the adhesion rates in tendons; for example, studies on flexor tendon repair showed that the Kessler suture can drastically reduce the likelihood of adhesion formation [6], which can be related to reduced friction due to fewer strands [53]. In pelvic surgery, recurrent curettage interventions seem to be the major risk factor for intrauterine adhesion formation after miscarriage [54]. Therefore, a surgical approach that reduces trauma to the endometrium is preferred to prevent adhesions. This approach would involve, for instance, reducing the employment of electrosurgery [55] or using smaller surgical tools for hysteroscopy [44].

Once adhesions have formed, surgery offers the possibility of excision with different techniques. However, as previously stated, the incremental number of procedures increases the odds of adhesion formation and poses an increased risk for the patient. Thus, techniques that prevent adhesion formation are preferred to recurrent interventions.

### Mechanical barriers

Mechanical barriers are widely used to prevent adhesions in different tissues, which has promoted the development of several related products for different tissue targets (Table 1). The principle of the use of mechanical barriers is to place a barrier around the surgical area that isolates it from surrounding tissues, avoiding the attachment of fibrin clots and further adhesion formation. Thus, a suitable mechanical barrier should be degradable, persist during the entire healing process and be inert to the immune response. Mechanical barriers can include solid polymers, gels and liquids [49].

**Table 1:** Commercial products available as mechanical barriers for different target tissues

Product	Type of product	Company	Target	FDA/CE approval	Clinical trial(s)
Seprafilm®	Solid barrier, hyaluronate carboxycellulose	Sanofi Genzyme, Massachusetts, USA	Peritoneum, pericardium, tendon	FDA, CE	Yes [49, 50] Yes [64, 65] No
Interceed®	Solid barrier, oxidised cellulose	Johnson & Johnson, New Jersey, USA	Peritoneum, endon	FDA, CE	Yes [51] No
Adept®	Liquid barrier, 4% icodextrin	Baxter, Illinois, USA	Peritoneum	FDA, CE	Yes [52–54]
SprayShield™	Liquid barrier, polyethylene glycol	Covidien-Medtronic, Minneapolis, USA	Peritoneum	CE	Yes [55–57]
Hyalobarrier®	Gel barrier, autocrosslinked hyaluronic acid	Anika Therapeutics, Bedford, USA	Peritoneum	CE	Yes [58]
REPEL-CV®	Solid barrier, ethylene oxide and polylactic acid	Pathfinder Cell Therapy (SyntheMed), Massachusetts, USA	Pericardium	FDA, CE	Yes [61]
CardioWrap®	Solid barrier, polylactic acid	MastBiosurgery AG, Zurich, Switzerland	Pericardium	FDA, CE	No
COVA™ + CARD	Solid barrier, collagen	Biom’up, Lyon, France	Pericardium	CE	Yes [67]
CorMatrix®	Solid barrier, porcine extracellular matrix	CorMatrix, Georgia, USA	Pericardium	FDA, CE	Yes [68]
Coseal™	Gel barrier, polyethylene glycol	Baxter, Illinois, USA	Pericardium	FDA, CE	Yes [72, 73]
Gore-Tex®	Solid barrier, expanded polytetrafluoroethylene	Gore & Associates, Delaware, USA	Pericardium	FDA, CE	Yes [74, 75]
Hyaloglide®	Gel barrier, autocrosslinked hyaluronic acid	Anika Therapeutics, Bedford, USA	Tendon	CE	Yes [78]
Adcon®Gel	Gel barrier, porcine gelatine and carbohydrate polymer	Bioscompass, Minnesota, USA	Tendon	CE	No
Tenoglide®	Solid barrier, collagen-GAG	Integra lifescience, New Jersey, USA	Tendon	FDA	No
DegraPol®	Solid barrier, polyester-urethane	DegraPol®, Lainate, Italy	Tendon	–	No

A considerable amount of work has been carried out to study different polymer materials of natural (i.e., hyaluronic acid, gelatin, collagen, phospholipids, cellulose, dextran or icodextrin) or synthetic (i.e., PLA, PEG, PGA, PCL or PVA) origin to produce barriers that have been assessed in multiple in vivo and clinical studies [56]. In addition, the combination and/or employment of mechanical barriers in different structures such as hydrogels, electrospun fibres, films or microspheres offers a wide range of possibilities that are being investigated [57, 58]. Recently, some studies have also investigated the use of tissue grafts, such as allogeneic amniotic membranes, in the prevention of adhesions with discouraging results in tendons and the peritoneum [59, 60] but more positive findings in the prevention of intrauterine adhesion recurrence [61, 62]. The features that a mechanical barrier must possess will depend on the surgical technique and the tissue that requires adhesion prevention.

In vivo and clinical trials addressing mechanical barriers are mostly focused on peritoneum and pelvic surgery, which accounts for a vast number of these studies [63, 64]. Currently, several products have been approved by European and United States authorities for use in abdominal and pelvic surgery, including Seprafilm® (hyaluronate carboxymethylcellulose), Interceed® (oxidised cellulose), Adept® (icodextrin 4%), Sprayshield™ (polyethylene glycol) and Hyalobarrier® (autocrosslinked polymers of hyaluronic acid). Although there was controversy regarding the safety of these products, new systematic reviews and clinical trials have demonstrated their safety but modest efficacy [63, 65–67]. Recently, Seprafilm® has been shown to reduce adhesions in a randomised clinical trial including 30 patients who underwent open abdominal surgery [68]. Seprafilm® has also shown effectiveness in major procedures (relaparotomy or Hartmann’s procedure) [69]. Similarly, Interceed® decreased adhesion formation (from 85.5 to 37.5%) in a clinical study including 38 patients who underwent reconstructive pelvic surgery [70]. However, in the case of pelvic and abdominal surgeries where laparoscopy is the most extended procedure, the application of gel and liquid barriers is easier than that of solid barriers, which are inappropriate for this technique [49]. Hence, the use of solid barriers is not approved for laparoscopy [8]. In intrauterine procedures, the use of a balloon, which prevents contact between denuded areas, is also an extended technique [55]. Regarding liquid or gel barriers, the safety of the antiadhesive solution Adept® was demonstrated a clinical trial involving 300 patients with small bowel obstruction [71]. In addition, a study in gynaecologic laparoscopy showed a reduction in the formation of adhesions in a clinical trial on 402 patients (10% reduction in the formation of de novo adhesions, *p = 0.029*) [72]. However, a more recent double-blinded randomised trial showed that Adept® had no clinical effect on adhesion formation, although it confirmed its safety [73]. Other adhesion barriers are available in Europe only, including Sprayshield™, which demonstrated effective reduction of the formation of adhesions [74, 75], although a previous work on adhesiolysis with recurrent adhesions showed its effectiveness in gasless laparoscopy only [76]. Similarly, in a small trial of 43 patients, Hyalobarrier® reduced the severity of adhesions, but there was no evidence regarding the reduction of the number of adhesion sites [77]. Other studies have been carried out with different mechanical barriers to battle peritoneal adhesions [78]. However, no definitive device has been developed yet.

Regarding the pericardium, several products are available to prevent adhesion formation after cardiac surgery. Among resorbable barriers, several different products are available. REPEL-CV® is a polymer film comprising ethylene oxide and lactic acid that is approved in Europe and the United States [79]. This product showed efficacy in reducing the incidence and severity of adhesions in a small study of paediatric cardiac surgery [80]. Furthermore, a preclinical study with dogs suggested that polylactic acid may serve as a scaffold for re-epithelisation, which would prevent the formation of adhesions [81]. CardioWrap®, another resorbable polylactide sheet available commercially, has been tested in preclinical studies. This solid adhesion barrier limited the formation of cardiac retrosternal adhesions in pigs [82]. Seprafilm® has also been proven to be safe in cardiac surgery and to reduce the formation of adhesions [83, 84]. Another barrier that consists of a collagen membrane is COVA™ CARD. Compared to Seprafilm®, this collagen barrier significantly reduced sternal adhesion formation in a preclinical study in sheep [85]. In addition, its safety and efficacy in reducing peritoneal adhesions has been proven recently in a clinical study of 65 patients [86]. Several clinical studies have also been carried out with the product CorMatrix®, a porcine decellularised ECM. Although these studies assessed only its safety and suitability for cardiac procedures and its capability to promote MSC differentiation towards cardiomyocytes in vivo [87], the retrosternal distance was maintained after 5 years [88], which may suggest the absence of sternal adhesions. Gel or liquid sealants are also employed for cardiac surgery as resorbable adhesion barriers where Coseal™ is the most extended. This polyethylene glycol gel decreased adhesion formation in preclinical [89, 90] and clinical [91, 92] studies. In the case of non-resorbable barriers, expanded polytetrafluoroethylene (ePTFE) Gore-Tex® was analysed as an adhesion barrier to prevent retrosternal adhesions after cardiac surgical procedures where it was linked with a reduction in the number of adhesions [93, 94]. Although substantial effort has been invested in adhesion prevention after cardiac procedures, modest progress has been achieved in the prevention of pericardial adhesions.

In relation to tendon adhesions, the barriers used to prevent them must also promote gliding to avoid interfering with tendon movement [95]. To this end, hyaluronic acid gels seem to be the most promising option, although positive results have been mostly observed in preclinical studies since the clinical findings have produced limited and moderate results [53, 96]. One example of a commercially available product based on hyaluronic acid is Hyaloglide®, a highly purified auto-cross-linked hyaluronic acid gel. Hyaloglide® was tested in a clinical trial on 45 patients undergoing tenolysis in zone II of the flexor tendon. Although the formation of recurrent adhesions was not assessed, Hyaloglide® showed a significant improvement in the range of motion and activity [97]. Adcon®Gel, a porcine gelatine combined with a carbohydrate polymer, is another gel barrier that showed promising results in the rabbit Achilles [98]. Solid barriers, such as Seprafilm® [99], Interceed® [100], Tenoglide® (a collagen-GAG matrix [101]) and DegraPol (an electro-spun polyester-urethane tube [102]), have also been assessed for tendons, mostly in preclinical models. However, preclinical studies of these products have resulted in promising but limited clinical data to support their use.

Overall, the success of mechanical barriers in the prevention of adhesions lays principally in their resorption time and mechanical stability. The duration must be long enough to act as an effective barrier during healing, but not long enough to trigger an inflammatory response and fibrotic deposition. In addition, these products require sufficient mechanical properties to facilitate their application and stability during and after surgery [56].

### Antiadhesive agents

Another front to battle adhesions is the use of adjuvants that interfere with the pathways that enhance the formation of adhesions or that promote those pathways that prevent their formation (Fig. 1). However, these pathways are complex and interconnected, which makes it challenging to find a definitive treatment to prevent the formation of adhesions. In fact, in the literature, the use of one extracellular mediator was suggested to be insufficient for the prevention of adhesion formation, whereas the use of multiple agents may have a synergetic effect [103, 104]. In addition, the absorption and diffusion properties of the mesothelium make it difficult to deliver these agents in a localised manner, especially in the peritoneum [78]. Another issue with these agents is their permanency and side effects during the healing process [105].

One of the earliest interventions was the employment of fibrinolytic agents. The discovery of the presence of fibrin during adhesion formation stimulated research on a variety of approaches to attack and resolve it. These initial studies included fibrinolysin, pepsin, trypsin, plasmin preparations and PA [7]. The basis of these agents is to promote fibrinolytic system activity or direct attacks on fibrin clots, preventing the origin of the formation of adhesions (Fig. 1). Currently, the main fibrinolytic agents investigated are streptokinase, t-PA and PAI-1. Nevertheless, the results obtained have shown poor performance in animal studies and side effects such as bleeding after their use [3, 78, 96, 105].

The control of local inflammation is another strategy that has been researched to develop antiadhesion agents. Inflammation is tightly associated with coagulation, fibrin deposition and consequently, with adhesions (Fig. 1). Thus, it is believed that a reduction in inflammation can attenuate adhesion formation. Hyaluronic acid has already been discussed as a mechanical barrier. However, hyaluronic acid must also be considered as an antiadhesive agent due to its anti-inflammatory properties and dissolutive effect on fibrin [56]. Although hyaluronic acid presents ideal properties as an antiadhesion material, the rapid resorption of hyaluronic acid represents a limitation for its use to prevent adhesion formation. Thus, strategies to extend its endurance in the body, such as crosslinking, are being studied to increase its antiadhesion properties. Other anti-inflammatory drugs have been tested in the peritoneum [78], pericardium [3] and tendon [96] to prevent adhesions. Examples of the studied agents include ibuprofen [106], celecoxib [107], resveratrol [108] or pirfenidone [109]. These agents target molecules that regulate the inflammation cascade, such as COX-1 and COX-2, in the case of non-steroidal anti-inflammatory drugs, or inflammatory cytokines, such as TGF-β or TFN-α, in the case of resveratrol or pirfenidone. Although the observed results seem promising in preclinical models, their employment as antiadhesive agents has not been evaluated clinically, and they pose an increase in the risk of undesired side effects [110].

Further strategies are based on the use of agents that limit cellular proliferation by preventing DNA replication, thus preventing fibroblasts from expanding and forming adhesions. Agents that present these characteristics include mitomycin-C, which has been demonstrated to reduce adhesions in a rabbit pericardial model [111], or 5-fluoroacil, which also reduced adhesions in the flexor tendon in chickens [112]. However, the side effects of these drugs still represent a crucial limitation for their use as antiadhesive agents.

Oestrogen is a hormone produced in the ovaries that plays a crucial role in endometrium development; thus, it could have a potential effect on the prevention of intrauterine adhesion formation. However, the use of oestrogen after hysteroscopy has not been demonstrated to provide significant positive effects [55]. Alternatively, oestrogen seems to be effective for the treatment of women with intrauterine adhesions, although combination with other systems that provide sustained release could improve patient outcomes [113].

Other methods, including anticoagulants (heparin), antioxidants (vitamin C) or neutralising antibodies for fibrinolytic inhibitors and inflammatory cytokines [114], have also been investigated. Although some of these agents showed positive results in animal studies, no conclusive data supporting their efficacy have been reported. Notwithstanding, the use of combined therapies that merge mechanical barriers and antiadhesive agents may support a promising approach to prevent the formation of adhesions in different surgeries. Some examples that have been investigated include the combination of Interceed® and heparin [115], which showed no improved efficacy with respect to any treatment alone in humans, or Seprafilm® combined with vitamin E, which showed similar results [116]. Other investigations have shown only limited results or modest improvements in animal studies [78].

Recent advances in molecular biology have also enabled new strategies adhesion prevention, where. Gene therapy represents a promising alternative or complementary approach. In the peritoneum, some examples of these strategies include the delivery of tPA genes to promote fibrinolysis with transgene viral vectors or the use of small interfering RNA (siRNA) to decrease the levels of hypoxic genes (HIF-1α) or decrease the action of fibrinolysis inhibitors (PAI-1) [114]. These strategies have shown moderate results. Similarly, the transfer of the HGF gene, which promotes mesothelial regeneration, by a viral vector showed a moderate reduction in peritoneal adhesions in a rat model [117]. Recent attempts in gene therapy that target adhesion formation in tendons have also employed adenoviral vectors [118] or antisense oligonucleotides [119] to inhibit the action of TGF-β with promising results. However, the presence of side effects indicates the need for a better understanding of the pathways where these molecular targets are involved.

The use of antiadhesive adjuvants offers great potential in the battle against adhesions, and their combination with mechanical barriers or sustained release platforms could enhance their effect and overcome their limitations. More research is needed to assess whether these agents are safe and efficient at preventing postsurgical adhesions alone or in combination with mechanical barriers. Particularly, more clinical trials are required to prove their safety and efficiency in different surgical procedures.

### Physical therapy

Physical therapy after surgery is a supplementary technique that can improve outcomes and reduce adhesion formation. In flexor tendon surgery, clinicians believe that the early motion of the digits prevents the formation of adhesions with adequate physical therapy; however, the state and strength of the tendon after surgery may limit the application of such therapy [120]. Early motion eliminates adhesions by physical contact due to the gliding of the tendon [121], preventing the settlement of adhesions and production of more fibrotic tissue.

Some studies in abdominal surgery indicate that manual therapy could be beneficial for adhesion prevention after surgery. Recently, in an in vivo study, Bove et al. showed that manual therapy after abdominal surgery attenuates the formation of adhesions in rats [122]. The authors suggested a mechanism similar to that in tendons; the motion of organs disrupts initially formed adhesions of deposited fibrin, preventing their settlement. Additionally, the authors showed a decrease in arginase and CD86 expression by macrophages in treated rats, suggesting the inhibition of the trophic switch of immune cells that subsequently inhibited the activation of fibroblasts. The inhibition of adhesions by visceral mobilisation was previously suggested by the same author [123]. In humans, manual therapy is employed as a conservative treatment for small bowel obstruction because it promotes its kinetics, but studies that prove the effect of physical therapy on adhesion prevention have been carried out in vivo only*.* Since the results obtained in vivo seem to prove that physical therapy is beneficial to prevent adhesions, it could represent a potential complementary treatment in clinics.

## Conclusion

Post-surgical adhesions still represent a major complication in most surgeries, with a particular impact on procedures in the peritoneum, uterus, pericardium and tendon where they may result in a serious setback for patients in terms of outcomes, causing pain, reoperation and tissue dysfunction. Adhesions occur due to an imbalance between fibrin deposition during coagulation and fibrin resolution directed by the fibrinolytic system where both systems maintain a tight relationship with inflammation. This imbalance is triggered by a disruption of the mesothelial/epithelial layer produced by surgery, irritation or inflammation.

Current research on therapies to prevent the formation of adhesions focuses on the use of mechanical barriers and antiadhesive adjuvants. Although serious efforts have been invested, limited positive results have been obtained in the prevention of adhesions, and these results have mostly been shown in animal models. Therefore, further efforts to understand and develop strategies against the formation of adhesions are needed. The use of combined strategies that involve mechanical barriers, adjuvants such as anti-inflammatories or hormones, and targeted gene therapy appears to be a promising option. To this end, carrying out blind randomised clinical trials is necessary to assess the safety and confirm the efficacy observed in animal trials of new therapies aimed at addressing the formation of postsurgical adhesions. In addition, the pursuit of new therapies must be synchronised with the development of effective surgical techniques that minimise the risk of their formation.

## Abbreviations

COX-1: Cyclooxygenase-1
COX-2: Cyclooxygenase-2
ECM: Extracellular matrix
ePTFE: Expanded polytetrafluoroethylene
GAG: Glycosaminoglycans
HGF: Hepatocyte growth factor
HIF-1α: Hypoxia-inducible factor 1α
IL-1: Interleukin-1
IL-10: Interleukin-10
IL-12: Interleukin-12
IL-1β: Interleukin-1β
IL-6: Interleukin-6
IL-8: Interleukin-8
MMP: Matrix metalloproteinase
MMP-1: Matrix metalloproteinase-1
MMP-3: Matrix metalloproteinase-3
MMP-9: Matrix metalloproteinase-9
MSC: Mesenchymal stem cells.
NAP-2: Neutrophil activating peptide-2
PA: Plasminogen activators
PAI: Plasminogen activator inhibitor
PAI-1: Plasminogen activator inhibitor-1
PAR: Plasminogen activator receptor
PCL: Polycaprolactone
PEG: Polyethylene glycol
PGA: Polyglycolic acid
PLA: Polylactic acid
PMC: Pericardial mesothelial cells
PVA: Polyvinyl alcohol
siRNA: Small interfering RNA
TGF-β: Transforming growth factor-β
TIMP-1: Tissue inhibitor of metalloproteinase-1
TNF- α: Tumour necrosis factor α
t-PA: Tissue plasminogen activator
u-PA: Urokinase plasminogen activator
u-PAR: Urokinase plasminogen activator receptor
VEGF: Vascular endothelial growth factor
